# Inhibition of LSD1 epigenetically attenuates oral cancer growth and metastasis

**DOI:** 10.18632/oncotarget.19637

**Published:** 2017-07-27

**Authors:** Saqer F. Alsaqer, Mustafa M. Tashkandi, Vinay K. Kartha, Ya-Ting Yang, Yazeed Alkheriji, Andrew Salama, Xaralabos Varelas, Maria Kukuruzinska, Stefano Monti, Manish V. Bais

**Affiliations:** ^1^ Department of Molecular and Cell Biology, Boston University Henry M. Goldman School of Dental Medicine, Boston, MA, USA; ^2^ Bioinformatics Program, Boston University, Boston, MA, USA; ^3^ Section of Computational Biomedicine, Boston University School of Medicine, Boston, MA, USA; ^4^ Department of Oral and Maxillofacial Surgery, Boston University Henry M. Goldman School of Dental Medicine, Boston, MA, USA; ^5^ Department of Biochemistry, Boston University School of Medicine, Boston, MA, USA

**Keywords:** LSD1, CTGF, OSCC, PDX, orthotopic mouse model

## Abstract

Lysine-specific demethylase 1 (LSD1) is a nuclear histone demethylase and a member of the amine oxidase (AO) family. LSD1 is a flavin-containing AO that specifically catalyzes the demethylation of mono- and di-methylated histone H3 lysine 4 through an FAD-dependent oxidative reaction. LSD1 is inappropriately upregulated in lung, liver, brain and esophageal cancers, where it promotes cancer initiation, progression, and metastasis. However, unlike other lysine-specific demethylases, the role and specific targets of LSD1 in oral squamous cell carcinoma (OSCC) pathogenesis remain unknown. We show that LSD1 protein expression was increased in malignant OSCC tissues in a clinical tissue microarray, and its expression correlated with progressive tumor stages. In an orthotopic oral cancer mouse model, LSD1 overexpression in aggressive HSC-3 cells promoted metastasis whereas knockdown of LSD1 inhibited tumor spread, suggesting that LSD1 is a key regulator of OSCC metastasis. Pharmacological inhibition of LSD1 using a specific small molecule inhibitor, GSK-LSD1, down-regulated EGF signaling pathway. Further, GSK-LSD1 attenuates CTGF/CCN2, MMP13, LOXL4 and vimentin expression but increased E-cadherin expression in pre-existing, patient-derived tonsillar OSCC xenografts. Similarly, GSK-LSD1 inhibited proliferation and CTGF expression in mesenchymal cells, including myoepithelial cells and osteosarcoma cells. In addition, gene set enrichment analysis revealed that GSK-LSD1 increased p53 expression and apoptosis while inhibiting c-myc, β-catenin and YAP-induced oncogenic transcriptional networks. These data reveal that aberrant LSD1 activation regulates key OSCC microenvironment and EMT promoting factors, including CTGF, LOXL4 and MMP13.

## INTRODUCTION

Histone demethylases play critical roles in oncogenesis [1, 2]. Lysine-specific demethylase 1 (LSD1) is a nuclear histone demethylase and a member of the flavin adenine dinucleotide (FAD)-dependent amine oxidase (AO) family that functions as an epigenetic regulator. AOs include diverse regulatory enzymes such as lysine-specific demethylases as well as the extracellular matrix lysyl oxidase enzyme family, serum amine oxidases, monoamine oxidases, and vascular adhesion protein-1 [3]. Lysine (K) methylation at specific histone positions—H3K9, H3K27, H3K37 and H4K20 is linked to the formation of tightly packed chromatin and gene silencing; in contrast, methylation of H3K4, H3K36 and H3K39 are associated with actively transcribed regions and gene activation [4]. LSD1 can demethylate H3K4 during gene repression and H3K9 during gene activation, indicating a dual and context-dependent role in transcriptional regulation. For example, LSD1 has a dual role in Notch signaling [5] and is both an activator and repressor of the androgen receptor in prostate cancer [6]. Further, LSD1 is inappropriately upregulated in lung, liver, brain and esophageal cancers through diverse regulatory mechanisms, including transcriptional activation and protein stabilization [7-11]. Inactivation of LSD1 promotes G1 arrest and induces differentiation-specific genes by selectively modulating methylation of H3K4 and H3K9 [12].

In particular, LSD1 promotes cancer initiation, progression and relapse through various mechanisms: supporting cancer initiating cells by increasing expression of the pluripotency-related genes SOX2, OCT4 and NANOG [12]; regulating expression of tumor suppressors such as E-cadherin and p53; and demethylating lysine residues of several non-histone substrates, including p53 [13], Dnmt1 (DNA (cytosine-5) methyltransferase [14] and E2F1 [15]. LSD1-mediated demethylation of H3K4 promotes Myc-induced transcriptional networks [16] and EGF signaling [17]. Importantly, these signaling pathways and genes are either mutated or upregulated in a variety of oral squamous cell carcinomas (OSCCs) [18]. Lastly, LSD1 is involved in epithelial-mesenchymal transitions (EMTs) known to contribute to metastasis of various cancers [19-21]. LSD1 functions in the NuRD nuclear protein complex and via that association contributes to the larger Co-REST complex [8]. Of note, the NuRD complex is recruited by a YAP/TAZ-TEAD complex to deacetylate histones [22]. Thus, an understanding of LSD1 functions will provide a foundation for better understanding epigenetic mechanisms underlying disease progression.

Oral cancer statistics are dismal, with 5-year survival rates of approximately 50 percent [23]. In this study, we investigated LSD1 expression to understand better how it may regulate oral cancer progression. We show that the LSD1 protein, unlike other known demethylases, is specifically deregulated during OSCC metastasis. Our data implicate LSD1 as a regulator of OSCC, highlighting its potential as a therapeutic target for future clinical applications.

## RESULTS

### LSD1 expression is aberrantly activated during OSCC progression

**Figure 1:**
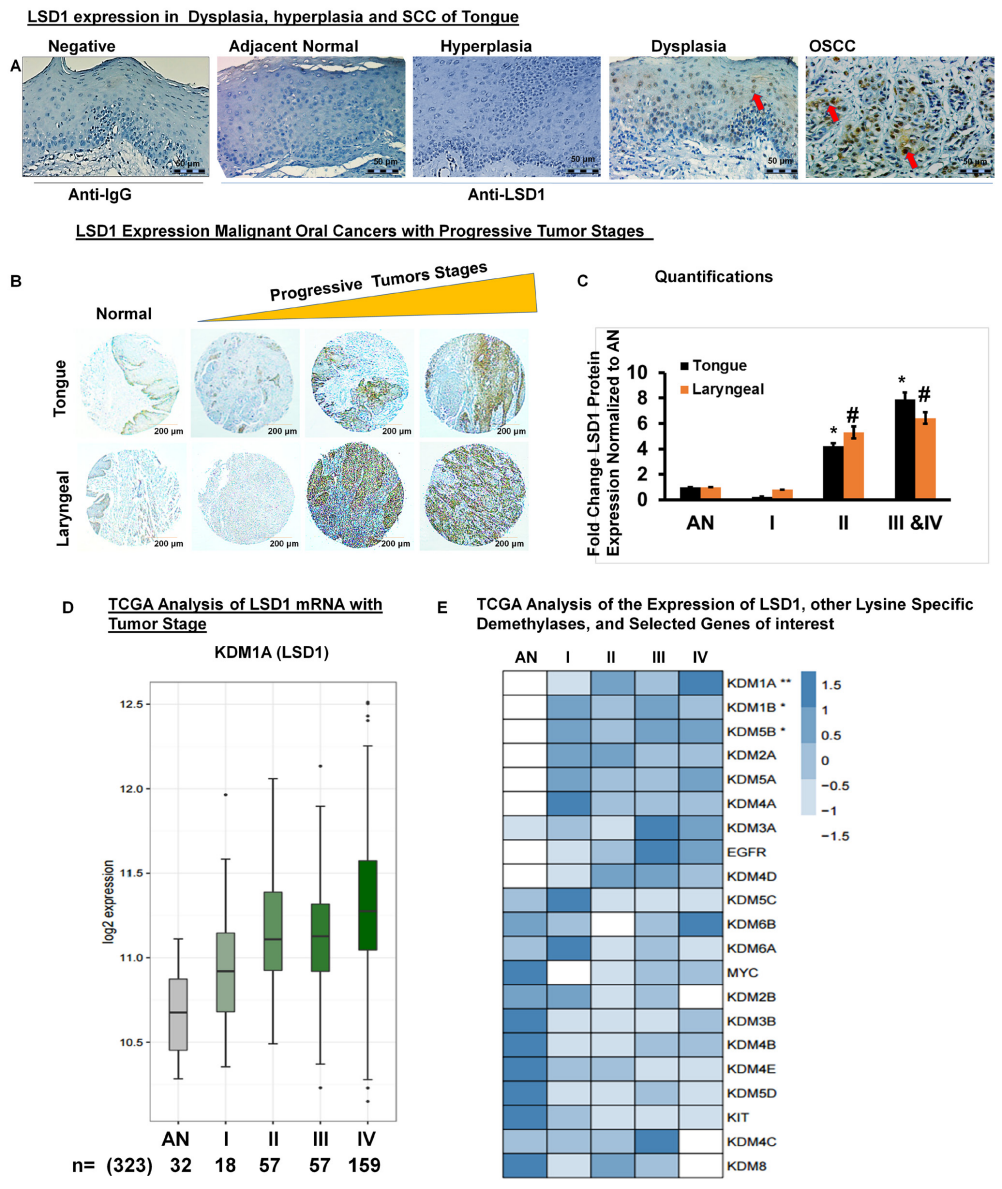
LSD1 is aberrantly expressed in OSCC **A.** LSD1 expression in adjacent normal tongue, hyperplastic, dysplastic and OSCC tissue evaluated by immunostaining; **B.** LSD1 expression in a human tumor microarray containing tissue from a diverse population of 80 malignant oral cancers; **C.** Quantification of LSD1 staining normalized to adjacent normal (AN) tissue; **D.** Data from The Cancer Genome Atlas (TCGA) showing the average LSD1 mRNA expression in progressive tumor stages (I-IV) compared to AN tongue tissue and **E.** A heatmap of the TCGA analysis of different tumor stages showing average expression levels (row Z-scores) for the 21 genes of interest (ranked in increasing order of the false discovery rate (FDR), ***FDR<0.0001, **FDR<0.01 and *FDR<0.05) with respect to expression of LSD1.

Immunohistochemistry for LSD1 performed on human clinical specimens representing normal adjacent, dysplastic, hyperplastic and OSCC tissues revealed that LSD1 expression is elevated in tumor tissues (Figure 1A). Next, we evaluated whether LSD1 expression increases during OSCC progression by using a tissue microarray containing a diverse population of 80 tumors of the larynx, tongue and submandibular gland as well as facial and other oral tumor types (Figure 1B). LSD1 staining was more intense in stage IIa- stage Iva tumors (Figure 1B and 1C). To determine if LSD1 is uniquely regulated compared to other histone demethylases, we performed mRNA expression analysis for OSCC using The Cancer Genome Atlas (TCGA) large dataset. We compared the mean expression of lysine-specific demethylase (KDM) genes to the expression of LSD1 (KDM1A mRNA) and additional selected cancer markers observed to be similarly regulated. We also assessed which of the known cancer drivers, KIT, EGFR or MYC, had the closest association with tumor progression. TCGA data analysis showed that LSD1 expression increases with tumor stage (Figure 1D) and tumor grade (Supplementary Figure 1A). TCGA data were also used to rank [using the false discovery rate (FDR)] the average expression levels (row Z-scores) of LSD1, other demethylases and selected genes of interest with respect to tumor stage (Figure 1E) and grade (Supplementary Figure 1). LSD1 gene expression was up-regulated significantly in OSCC stage II and IV tumors. However, for the samples available in the TCGA database, LSD1 expression in stage III tumors was not progressively increased. Thus, the histological and bioinformatic analyses demonstrated that LSD1 is an important regulator of OSCC.

### LSD1 overexpression promotes OSCC metastasis

**Figure 2:**
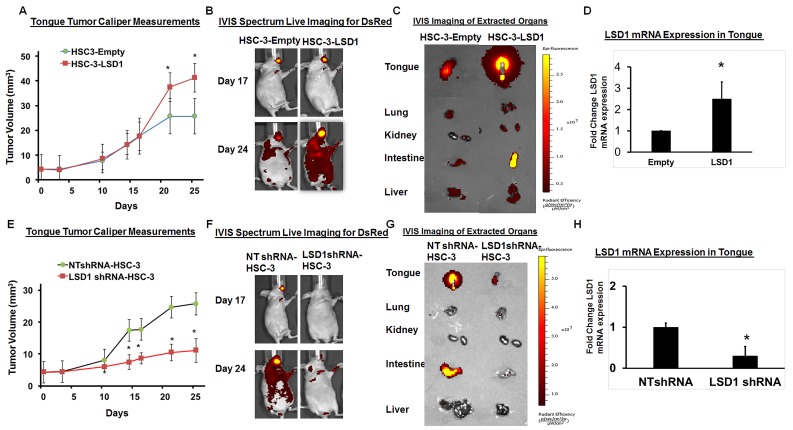
LSD1 overexpression promotes OSCC growth and metastasis **A.** HSC-3-LSD1 cells implanted into the tongue of nude mice (*n* = 5 per condition) grew more than HSC-3-control cells, as evaluated by caliper measurements; **B.** An *in vivo* imaging system (IVIS) revealed that on day 17 post-implantation, tumors derived from HSC-3-LSD1 and HSC-3-control cells appeared similar whereas by day 24, the tumors derived from HSC-3-LSD1 cells displayed increased growth and metastasis; **C.** IVIS imaging of extracted internal organs demonstrating tongue tumor growth and metastasis and **D.** RT-qPCR analysis of LSD1 expression. **E.** ShLSD1-HSC-3 cells implanted into the tongue of nude mice (*n* = 8 per condition) grew dramatically less than HSC-3-control cells, as evaluated by caliper measurements; **F.** IVIS on day 17 post-implantation revealed only a slight difference in HSC-3-LSD1-derived tumors compared to HSC-3-derived tumors whereas by day 24, HSC-3-LSD1 tumors showed reduced growth and metastasis; **G.** IVIS imaging of extracted internal organs demonstrating tongue tumor growth and metastasis and **H.** RT-qPCR analysis of LSD1 expression. Statistical analyses were performed with unpaired Student’s t-tests. * P-value<0.05.

To evaluate the physiological effect of LSD1 overexpression on OSCC growth and metastasis, we established HSC-3 cells overexpressing LSD1 and tested them in our previously described oral cancer orthotopic nude mouse model [24-27]. HSC-3-LSD1 cells implanted into the tongue showed extensive growth and metastasis compared to HSC-3-control cells (Figure 2A-2D). Caliper measurements of tongue tumors derived from HSC-3-LSD1 cells showed a progressive increase in tumor volume (Figure 2A). *In vivo* imaging studies (IVIS) showed increased tumor growth and metastasis at day 24 (Figure 2B and 2C). Thus, LSD1 overexpression induced an aggressive phenotype in HSC-3 cells *in vivo*. To evaluate the effect of LSD1 loss of function, HSC-3 cells were stably integrated with HSC-3-shLSD1 or NTshRNA (Figure 2E-2H). HSC-3-shLSD1 cells implanted into the orthotopic OSCC model showed a significant reduction in tumor growth, as indicated by a reduction of tongue tumor volume in caliper measurements, and a reduction of metastasis, as indicated by IVIS visualization on day 24. The knockdown of LSD1 inhibited tongue tumor growth and metastasis to internal organs. Thus, overexpression of LSD1 promotes HSC-3-induced tumor growth and metastasis, whereas loss of LSD1 is inhibitory. These data establish that LSD1 is an important driver of OSCC in this orthotopic model.

### Pharmacological inhibition of LSD1 with GSK-LSD1 attenuates oncogenic properties in metastatic HSC-3 cells

**Figure 3:**
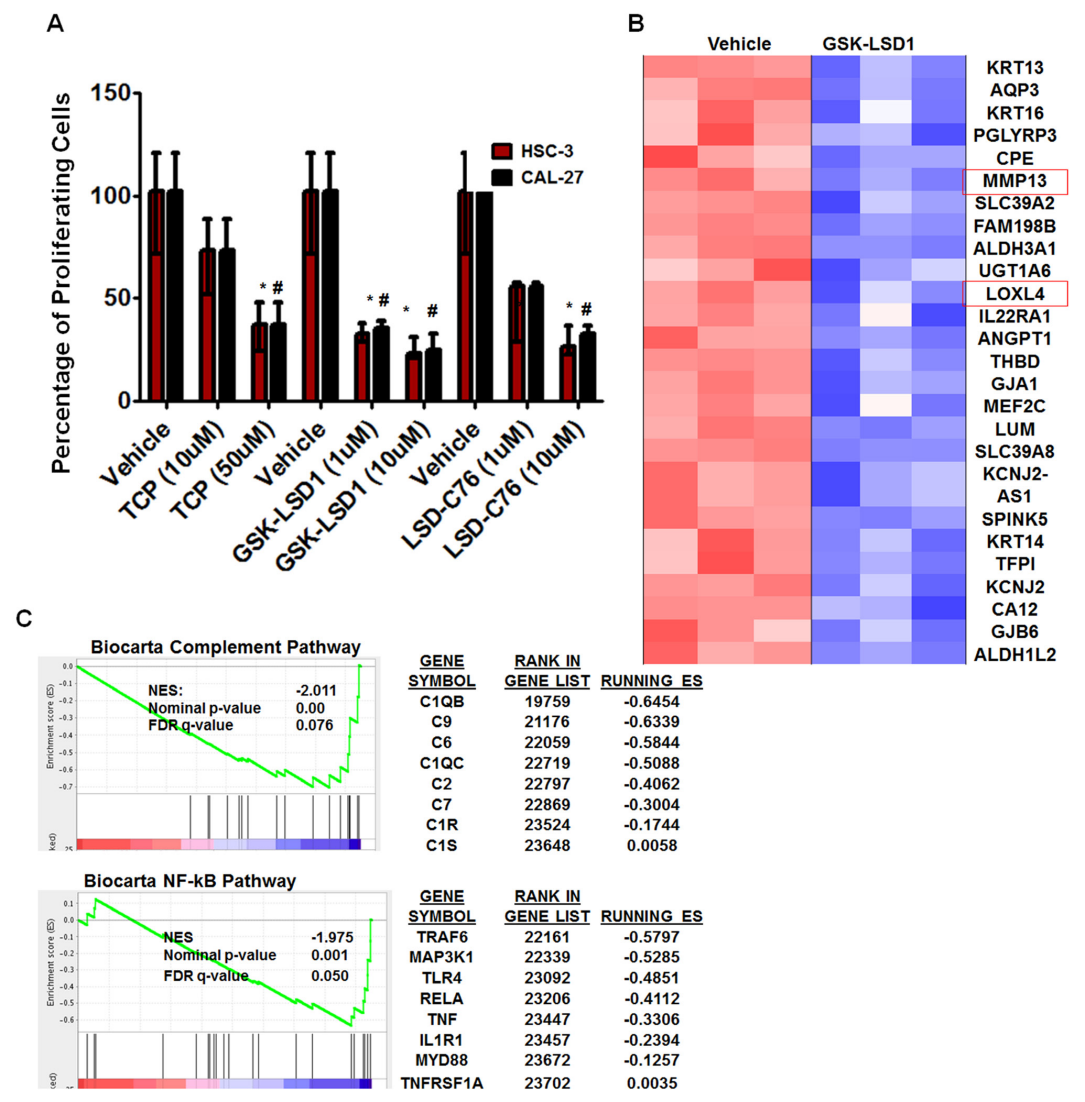
An LSD1 inhibitor (GSK-LSD1) attenuates proliferation and inhibits key targets in HSC-3 cells **A.** Inhibition of LSD1 by different inhibitors (*n* = 6 replicates per treatment), including tranylcypromine (TCP), GSK-LSD1 and LSD1-C76 impaired the proliferation of HSC-3 and CAL-27 cells; **B.** Microarray and gene set enrichment analysis (GSEA) (*n* = 3 replicates per treatment) revealed that GSK-LSD1 inhibits key genes and **C.** GSEA indicated that GSK-LSD1 inhibits specific signaling pathways. Statistical analyses were performed with unpaired Student’s t-tests. The significant differences are indicated with * P-value<0.05 in HSC3 cells and ^#^P-value<0.05 in CAL-27 cells.

To further characterize the role of LSD1 in OSCC, we evaluated 3 different inhibitors of LSD1: tranylcypromine (TCP), which is a non-selective LSD1 inhibitor that also inhibits monoamine oxidases A and B; and GSK-LSD1 (GlaxoSmithKline) and LSD1-C76 (Xcessbio), which are selective LSD1 inhibitors with 1,000-fold more selectivity for LSD1 than its other isoforms. TCP, GSK-LSD1 and LSD-C76 inhibited proliferation of HSC-3 and CAL-27 cells, but GSK-LSD1 was the most effective inhibitor at 1 µM concentration for both HSC-3 and CAL-27 cells and was therefore used for subsequent studies (Figure 3A).

Microarray analysis from three biological replicates showed that GSK-LSD1 inhibited key genes involved in OSCC growth and metastasis (arranged by lowest to highest FDR value) (Figure 3B). Moreover, gene set enrichment analysis (GSEA) showed that GSK-LSD1 inhibited the complement cascade, NF-kB, and other inflammatory pathways known to be critical in cancer progression and metastasis (Figure 3C).

### GSK-LSD1 attenuates EGF-induced proliferation and signaling

**Figure 4:**
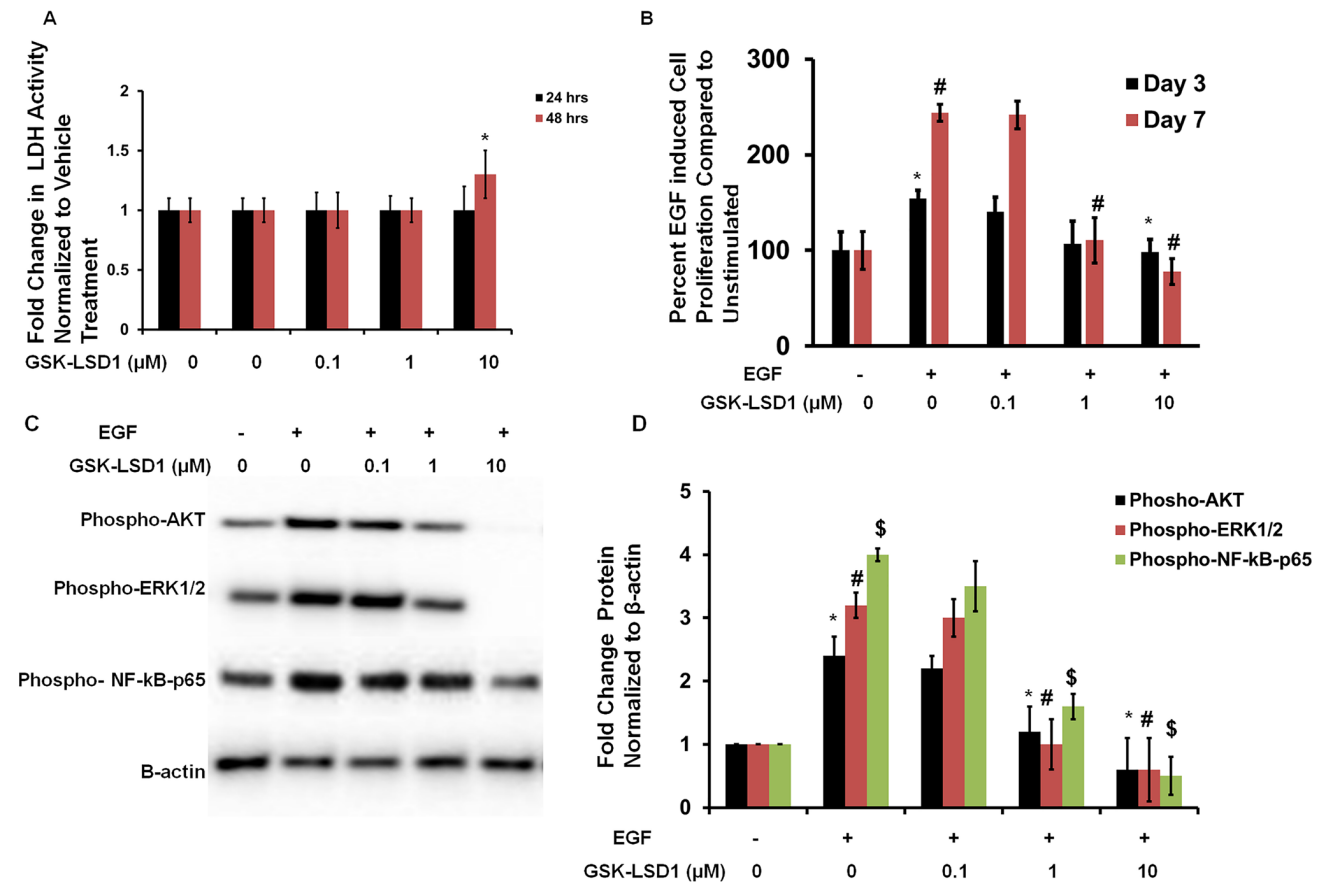
GSK-LSD1 attenuates EGF signaling **A.** LDH activity in HSC-3 cells treated with different concentrations of GSK-LSD1 at 24 and 48 hours; **B.** EGF-induced proliferation in HSC-3 cells is inhibited by GSK-LSD1; **C.** GSK-LSD1 inhibits phospho-AKT, -ERK1/2 and -NF-κB-p65 in HSC-3 cells and **D.** quantitation of this inhibition. Statistical analyses were performed with unpaired Student’s t-tests. The significant differences are indicated with ^*, # or $^ P-value<0.005 in respective groups.

To evaluate cytotoxicity and any non-specific effects of GSK-LSD1, lactate dehydrogenase (LDH) assays were performed. LDH activity was measured at 24 and 48 h in HSC-3 cells treated with different concentrations of GSK-LSD1. The 0.1 and 1 μM doses did not affect LDH release. However, the 10 μM dose increased LDH activity at 48 h (Figure 4A). Additionally, the EGF-induced proliferation of HSC-3 cells was inhibited by 1 and 10 μM GSK-LSD1 (Figure 4B). Molecular signaling analysis showed that GSK-LSD1 inhibits phospho-AKT, -ERK1/2 and -NF-κB-p65 in HSC-3 cells (Figure 4C and 4D). Thus, GSK-LSD1 inhibits EGF-induced signaling and proliferation without cytotoxicity in oral cancer cells. This represents a potential mechanism for the inhibitory effects of GSK-LSD1.

### Blocking aberrant LSD1 activity down-regulates expression of CTGF, LOXL4, MMP13 and vimentin in pre-existing tonsillar OSCC patient-derived xenograft (PDX) mouse models

**Figure 5:**
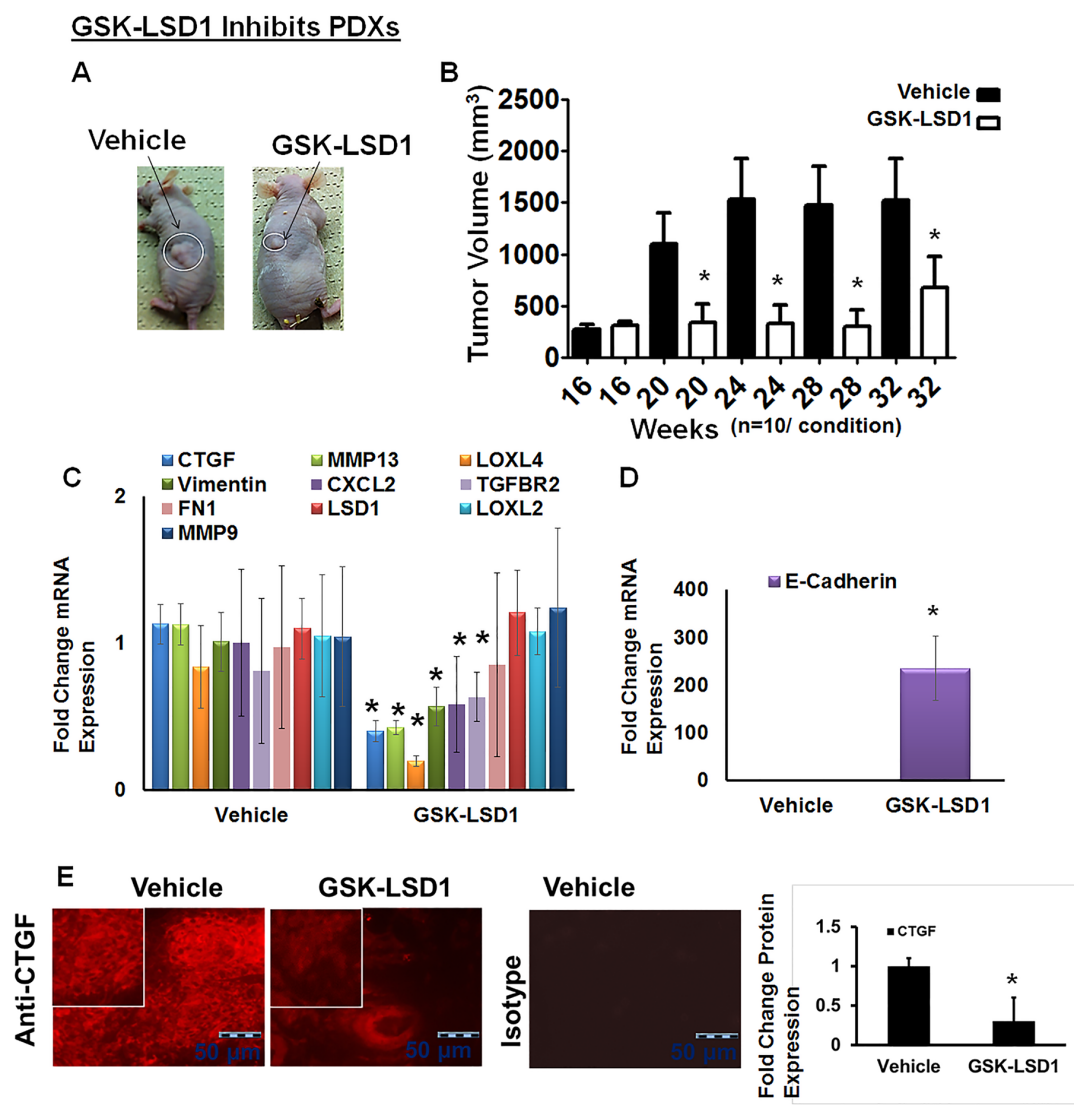
Blocking aberrant LSD1 affects expression of CTGF, LOXL4 and MMP13 in a pre-existing tonsillar OSCC patient-derived xenograft model (PDX) **A.** GSK-LSD1 injection inhibits the growth of pre-existing tumors as detected at 32 weeks in a tonsillar OSCC PDX model (*n* = 10 per condition). Statistical analyses were performed with unpaired Student’s t-tests. * P-value<0.05. **B.** Caliper measurements of tumor volumes; **C.** GSK-LSD1 reduced the mRNA expression levels of CTGF, MMP13, LOXL4, and Vimentin expression; **D.** GSK-LSD1 increased the expression of E-cadherin and **E.** Relative to vehicle injection, GSK-LSD1 injection inhibits CTGF expression in pre-existing tonsillar OSCC PDX tissue sections. Statistical analyses were performed with unpaired Student’s t-tests. * P-value<0.05.

We have established unique PDX models in which implantation of 5-mm tumor explants generates tonsillar tumors at 4 months with an average size of 293 mm^3^. Tonsil SCCs are semisolid cystic tumors, which develop lymph inside the tumor once they start growing in nude mice. Biweekly injection of GSK-LSD1 (10 mg/kg) into animals with xenografts grown for 16 weeks inhibited further xenograft growth, with tumors in inhibitor-injected mice showing a reduction in size by 32 weeks; tumors in vehicle-injected control mice grew significantly larger, reaching volumes up to 3,000 mm^3^ (Figure 5A and 5B). GSK-LSD1 inhibited tonsillar SCC patient-derived tumor xenografts and inhibited CCN2/CTGF, MMP13, LOXL4 and vimentin expression whereas the expression of the tumor suppressor E-cadherin increased (Figure 5C and 5D). Further, GSK-LSD1 inhibited CTGF expression in PDXs (Figure 5E). Biweekly injection of GSK-LSD1 (10 mg/kg) starting at 20 weeks, when tumor growth was maximal, inhibited further tumor growth (Supplementary Figure 2). However, inhibitor injections post-20 weeks were not as efficient possibly due to the larger sizes of the pre-existing tumors. Thus, GSK-LSD1 inhibits the growth of pre-existing tumors in a PDX model possibly by down-regulating CCN2/CTGF, MMP13, LOXL4 and vimentin expression and restoring E-cadherin function.

### Pharmacological inhibition of LSD1 attenuates distinct oncogenic transcriptional networks

**Figure 6:**
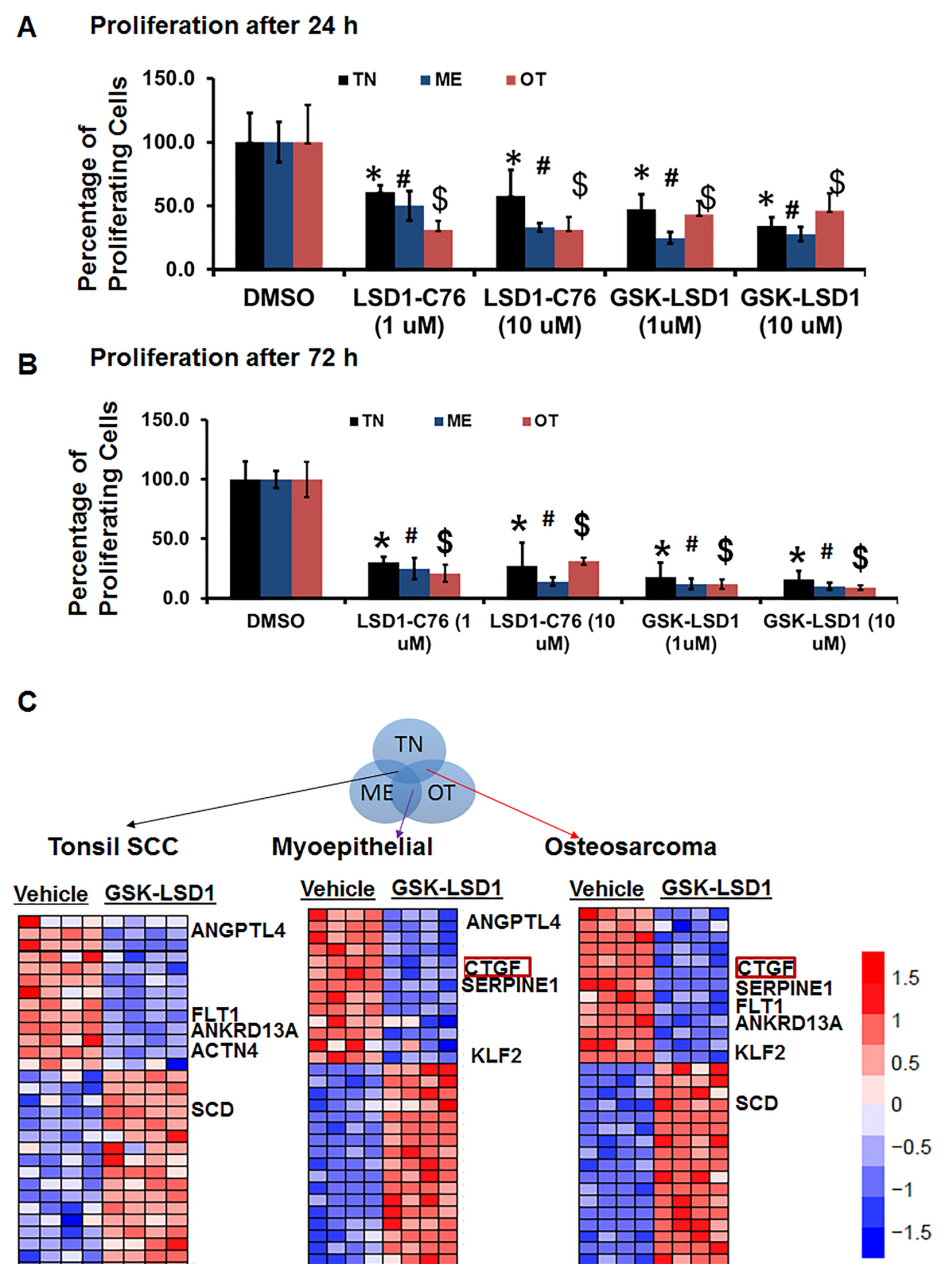
Pharmacological inhibition of LSD1 inhibits CTGF and metastatic OSCC mediators Patient-derived oral cancer primary cells were used to evaluate the effectiveness of GSK-LSD1 because drugs tested on cell lines such as HSC-3 and CAL-27 may not be effective on primary tumor cells, and there may be differences in molecular targets and signaling pathways. The most common SCC in OSCC arises at the base of the tongue and tonsil. First, we evaluated tonsil SCC primary cells (TN, from 1 patient with 4 biological replicates) to evaluate key targets and signaling pathways regulated by GSK-LSD1. Then, we extended our observations to myoepithelial (ME, from 1 patient with 4 biological replicates) primary tumors which represent 1-2% of OSCCs and have both epithelial and mesenchymal populations, and osteosarcomas (OT, from 1 patient with 4 biological replicates) which are very rare and of mesenchymal origin. **A.** LSD1 inhibition prevented the proliferation of patient-derived OSCC tumor cells at 24 h; **B.** LSD1 inhibition prevented the proliferation of patient-derived OSCC tumor cells at 72 h and **C.** Selected gene signatures showing similarity in genes inhibited or activated by GSK-LSD1 compared to vehicle in patient-derived primary cells isolated from clinical tumors. Statistical analyses were performed with unpaired Student’s t-tests. Statistical analyses were performed with unpaired Student’s t-tests. The significant differences are indicated with ^*, # or $^ P-value<0.005 in respective groups.

**Figure 7:**
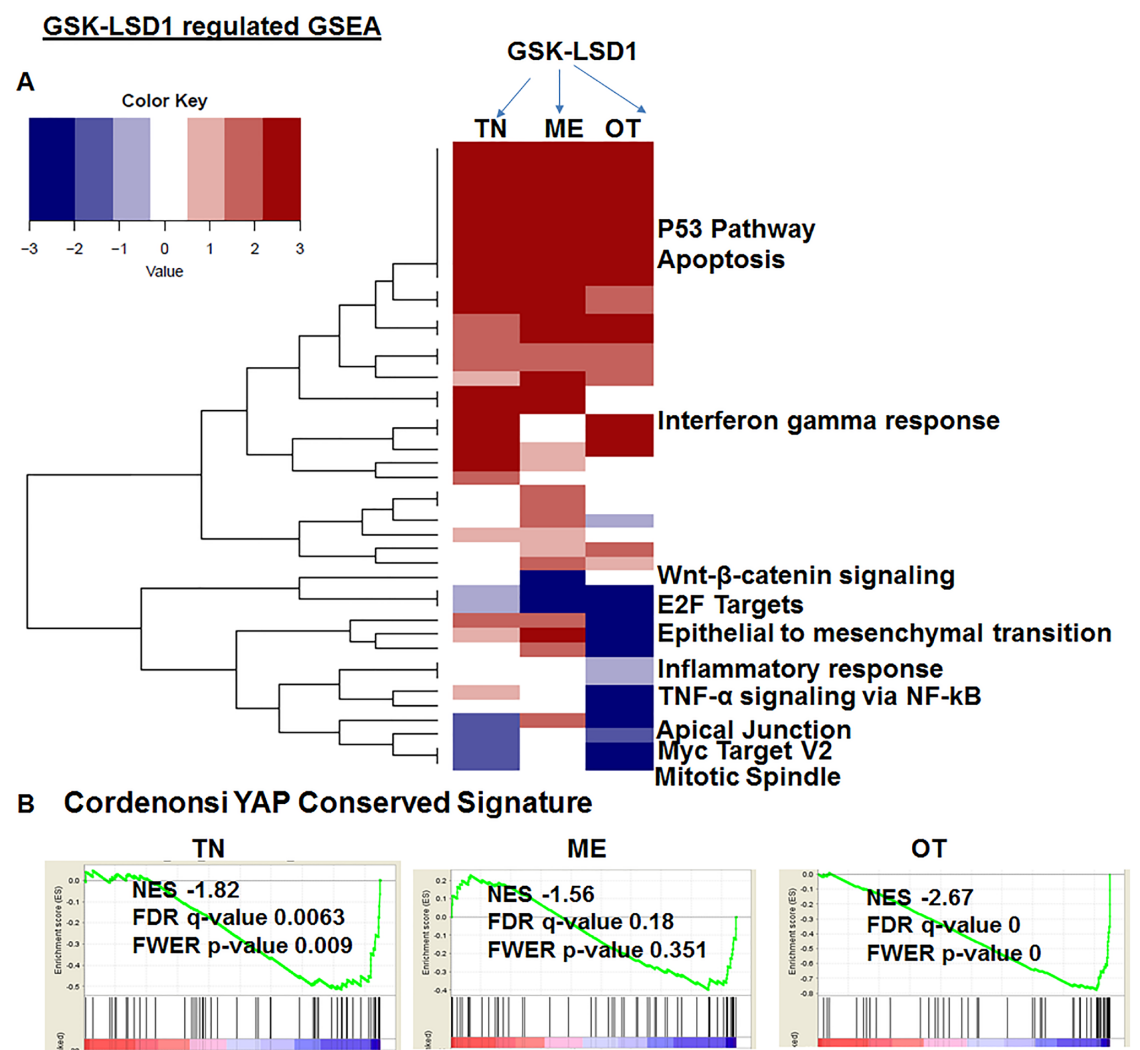
Pharmacological inhibition of GSK-LSD1 inhibits a transcriptional signaling network that induces the expression of CTGF and metastatic OSCC mediators **A.** A heatmap of signaling pathways which are activated or repressed; the values plotted in the heatmap are based on the signed FDR values resulting from the GSEA analysis of GSK-LSD1 treatment vs control treatment with respect to hallmark gene sets (*n* = 50) and **B.** Comparative analysis of YAP1-induced GSEA in 3 different primary cell types.

Because studies performed with HSC-3 and CAL-27 cell lines do not to reflect the heterogeneity of tumors, we examined patient-derived primary cells/explants isolated from clinical specimens. These patient-derived tumor cells are likely to mimic the tumor microenvironment since they contain a diverse population of cells enriched with cancer cells. GSK-LSD1 treatment of primary patient-derived cells inhibited the growth of tonsil SCC (TN), myoepithelial (ME) and osteosarcoma (OT) cells. These primary cells have different origins, but after 24 (Figure 6A) and 48 h (Figure 6B), treatment with 1 and 10 µM of the LSD1 specific inhibitors LSD1-C76 (Xcessbio) and GSK-LSD1 (GSK) attenuated their proliferation. Next, microarray analysis demonstrated that inhibition of LSD1 inhibited common differentially expressed genes related to tumor growth and metastasis (Figure 6C). The common gene signature in cells derived from 3 different patients revealed that GSK-LSD1 inhibited CTGF, FLT1 (VEGFR1), ANGPTL4, Serpine 1 and FBN2 (Figure 6C). A heatmap summarizing hallmark gene set enrichments across all three tumor cell types is shown in Figure 7A. Several gene sets were up-regulated across all three tumor-derived isolates, including the p53 pathway and other pro-apoptotic pathways. This is also supported by our data in Figure 4 that GSK-LSD1 inhibits EGF-induced proliferation and signaling pathways. Additionally, Wnt/β-catenin signaling was significantly down-regulated upon GSK-LSD1 treatment in ME cells, while no correlation with GSK-LSD1 treatment was observed in the other two cell types for this pathway. GSEA also showed inhibition of YAP1-, E2F1-, and myc-induced signatures. Although the YAP-induced transcriptional network [28] was inhibited in TN, ME and OT cells, there were differences in the specific genes that were enriched (Figure 7B). Thus, the GSEA analysis identified similarities and some unique differences in the inhibition of specific gene signatures by GSK-LSD1, suggesting that the effects of LSD1 were tumor context-dependent.

### GSK-LSD1 restores global dimethylation

**Figure 8:**
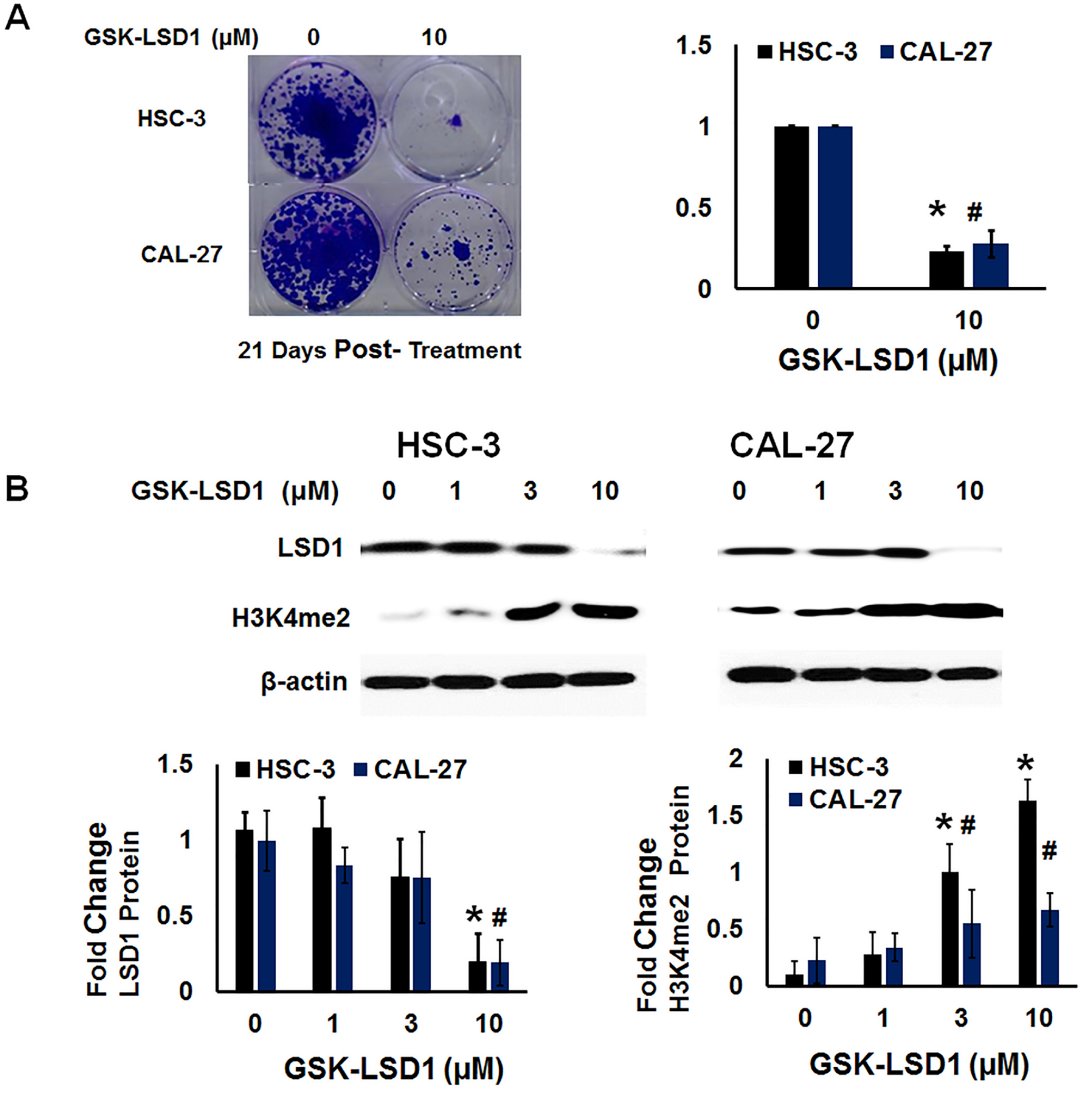
LSD1 inhibition restores dimethylation and inhibits clonogenic survival of non-metastatic CAL-27 and metastatic HSC-3 cells *in vitro* **A.** Clonogenic survival was inhibited more than 70% in HSC-3 and CAL-27 cells treated with GSK-LSD1 and **B.** GSK-LSD1 reduced LSD1 protein expression and restored dimethylation of histone H3 (H3K4me2). Statistical analyses were performed with unpaired Student’s t-tests. The significant differences are indicated with ^* or #^ P-value<0.05 in respective groups.

We performed functional analysis of expression of H3K4me2 and clonogenic survival (Figure 8A and 8B) after treatment with GSK-LSD1. GSK-LSD1 inhibited clonogenic survival by more than 70% in HSC-3 and CAL-27 cells (Figure 8A). As shown in Figure 7B, GSK-LSD1 inhibited LSD1 protein expression while restoring levels of dimethylated histone H3 (H3K4me2). Thus, pharmacological inhibition of LSD1 inhibited expression of key genes and signaling networks leading to reduced cell proliferation and clonogenic survival while restoring H3K4me levels. Collectively, these results align changes in LSD1 expression with histone methylation status and with functional outcomes.

## DISCUSSION

Locoregional spread and metastasis to lymph nodes are the leading causes of poor patient outcomes in OSCC. Treatment for OSCC frequently involves a combination of surgery, radiotherapy, and chemotherapy. However, resistance to therapy is a challenge, and the average 5-year survival rate remains approximately 50%. Here, we show that one of the mechanisms underlying the aggressive features of OSCC is aberrant activation of LSD1. We align LSD1 expression with demethylation of H3K4me2 and with inappropriate activation of the YAP1-CTGF axis and LOXL4 expression. Importantly, our studies suggest that pharmacological inhibition of LSD1 is likely to attenuate aggressive OSCC.

LSD1 regulates the balance between H3K4 and H3K9 dimethylation with H3K27 trimethylation, or between active chromatin, heterochromatin, and repressed chromatin states [29, 30]. LSD1 demethylates histone and non-histone genes by removing mono- and dimethyl groups from histone H3 at lysine 4 (H3K4me1/2). LSD1 is a potential target of metastatic OSCC therapy, as suggested by this study which demonstrates that LSD1 expression is increased in human malignant OSCCs of the oral cavity and that its expression tracks with progressive staging.

This study provides evidence that GSK-LSD1 regulates EGF-mediated signaling pathways and network. LSD1 regulates orthotopic oral cancer metastasis *in vivo* (Figure 2). OSCCs are of epithelial origin and their locoregional spread and metastasis are likely to rely on the EMT process. We used different tumor types including epithelial tonsil SCC (TN) which is abundant in head and neck squamous cell carcinoma (HNSCC); myoepithelial tumors (ME), which constitute between 1-2% of total OSCC and have a combined epithelial and mesenchymal origin; and osteosarcoma (OT), a very rare tumor of mesenchymal origin. GSK-LSD1 inhibits the EMT-related target gene vimentin and up-regulates E-cadherin. Small molecule specific inhibitors of LSD1 have been developed by GlaxoSmithKline (GSK1-LSD1; GSK2879552) [31] and they are in phase 1 clinical trials for lung cancer. Previous studies have shown that Pargyline, a nonspecific inhibitor of LSD1, interfered with the EMT of prostate cancer *in vivo*, and the lysine-specific demethylase UTX inhibited EMT-induced breast cancer stem cell properties by epigenetic repression of EMT genes in cooperation with LSD1 and HDAC1 [32, 33]. GSK-LSD1 inhibited CTGF and other oncogenic factors in TN, ME and OT primary patient-derived cells in vitro (Supplementary Figure 3). Here, we show that pharmacological inhibition of LSD1 inhibits the aggressive features of OSCC by inhibiting key target genes that function in the tumor microenvironment and EMT, including CCN2/CTGF, MMP13, LOXL4 and vimentin.

GSEA analysis showed that pharmacological inhibition of LSD1 abrogated the EGF and YAP1 signaling network. Previous studies have shown that CTGF promotes EGF signaling, the central signaling pathway in OSCC [34]. Since LSD1 can mediate EGF signaling [17] during oncogenesis, we conclude that inhibition of LSD1 can attenuate CTGF, an activator of EGF signaling in aggressive OSCC. The GSK-LSD1 regulated hallmark and oncogenic signaling networks are provided in Supplementary Tables 1 and 2.

This study is the first to demonstrate that pharmacological inhibition of LSD1 in OSCC and other cancer types attenuates the YAP1 oncogenic pathway. CTGF and YAP collaborate to promote tumor growth and metastasis, and CTGF itself is a downstream target of YAP [35, 36]. Further, CTGF is a downstream effector of LSD1, which is itself a component of the NuRD co-repressor complex [8] recruited by the YAP/TAZ-TEAD complex to deacetylate histones and alter nucleosome occupancy at target genes [22]. Thus, LSD1 and YAP1 are also likely to collaborate to induce CTGF activation in OSCC.

In conclusion, our data reveal that 1) LSD1 is aberrantly activated in metastatic OSCC, and increases in LSD1 expression correlate with advanced disease; 2) LSD1 overexpression increases metastatic OSCC in orthotopic oral cancer mouse models; 3) pharmacological inhibition of LSD1 attenuates ECM and EMT-related genes such as CTGF, LOXL4, MMP13 and vimentin and increases E-cadherin expression in patient-derived pre-existing xenografts and cellular models; 4) GSK-LSD1 inhibits EGF-induced signaling and proliferation without cytotoxicity in oral cancer ; and 5) LSD1 inhibition is likely to regulate the EGF and YAP1 signaling network. Thus, our findings have potential prognostic and therapeutic application in the clinical management of OSCC.

## MATERIALS AND METHODS

### Human tissues and animal experimental approval

For experiments using human tissue, informed consent was obtained from patients at the Boston University Medical Center. Mouse experiments were reviewed and approved by the Institutional Animal Care and Use Committee (IACUC #AN-15390).

### LSD1 immunostaining

Normal, dysplastic, hyperplastic and oral SCC tissue sections were stained with the anti-LSD1 antibody (Abcam: ab17221). Similarly, a tissue microarray (US Biomax, HN802a), which has 80 tissue samples representing different tumor grades and stages was stained with the anti-LSD1 antibody (Abcam: ab17221,1 µg/ml). Quantitation of staining was performed using Image J software (NIH) with the IHC profiler plugin for DAB immunostaining analysis [37]. Each image from the tissue microarray was deconvoluted in the IHC profiler plugin to obtain different measurements for DAB and hematoxylin. Parameters were set to measure the mean, min, and max intensities for stained regions, and data were obtained from several regions. The log value of the average max intensity was calculated. The fold changes in expression compared to respective controls (AN) are indicated in the graph.

### Expression analysis of The Cancer Genome Atlas OSCC data

RNA-sequencing (RNASeq) and matched clinical data corresponding to primary human OSSC samples from The Cancer Genome Atlas (TCGA) were obtained as previously described [38]. Box plots of the expression values (log2-transformed) for genes of interest were generated with respect to tumor grade, after removing samples with missing grade information. Positive association of the expression of select genes with respect to tumor progression was tested by comparing adjacent tumor grades (i.e. grade 2 vs grade 1, grade 3 vs grade 2, and grade 4 vs grade 3) using a one-tailed t-test, and the p-values combined per gene as previously described [39]. P-values were adjusted for multiple hypothesis testing across all genes being compared using the false discovery rate (FDR) to obtain the reported q-values. The association of LSD1 (KDM1A) compared to other lysine-specific demethylases and histological tumor grade and stage from TCGA oral cancer RNAseq datasets of 343 OSCC specimens was also evaluated.

### In vitro assays

Proliferation assays were performed by plating HSC-3 cells and CAL-27 cells (20,000 cells per well, 6-replicates per treatment) overnight. The next day, cells were treated for 48 h with a vehicle control, or the LSD1 inhibitors tranylcypromine (TCP), GSK-LSD1 or LSD-C76 and the proliferation percentage was evaluated by the CyQuant assay. The effect of EGF on proliferation was evaluated using HSC-3 cells (20,000 cells per well, 6 replicates per treatment) and was evaluated by the CyQuant assay at different time points. EGF-induced signaling pathways were evaluated in HSC-3 cells (200,000 cells per well) and evaluated by Western blot analysis as previously described [40, 41]. LDH based cytotoxicity assays were performed on HSC-3 cells (20,000 cells per well, 6-replicates per treatment) per the manufacturer’s instructions (Promega, Inc) [42]. To determine the effect of LSD1 inhibition on clonogenic survival, a total of 500 HSC-3 or CAL-27 cells were plated and incubated for 24 h with 0 or 10 µM GSK-LSD1, allowed to grow for 21 days, then stained with 0.1% crystal violet.

### Orthotropic OSCC mouse model

Short Tandem Repeat based authentication of HSC-3 cells were performed by Cell Line Authentication Services (Genetica DNA Laboratories). The orthotopic OSCC mouse model was established by implantation of HSC-3-DsRed cells to evaluate OSCC growth and metastasis. The details of this protocol were published previously [27]. To determine the effect of LSD1 inhibition *in vivo*, HSC-3- shLSD1 and HSC-3- NTshRNA cells were prepared by transducing DsRed expressing HSC-3 cells with shLSD1 or NT-shRNA lentivirus particles, respectively, and implanted into the tongues of nude mice [27]. Tumor growth and metastasis were evaluated by tongue tumor caliper measurements and live *in vivo* imaging using the IVIS twice a week for 4 weeks post-implantation. Next, to evaluate the effect of LSD1 overexpression, HSC-3 DsRed cells were infected with PLX-304-CMV-LSD1-V5 or PLX-304-CMV-Empty-V5 lentivirus particles to generate HSC-3 LSD1 and HSC-3 control cells, respectively. HSC-3 control and HSC-3 LSD1 cells were injected into the tongues of mice (*n* = 5 per condition), and the mice were monitored for tongue tumor growth by caliper measurements and the IVIS twice a week for 4 weeks.

### Patient-derived head and neck cancer cells

Cells were derived from patients with recurrent tonsillar epithelial, myoepithelial and osteosarcoma tumors. Freshly isolated tumor tissues were resected into 0.5 mm pieces and grown in the presence of reduced serum and growth factors. Primary tumor cells were trypsinized along with the remaining small tissue explant and used in different experiments. Cells from the same preparations were also tested in an orthotopic patient-derived mouse model for the formation of tumors. Of note, the tonsil SCC from a non-smoker was p16 + as evaluated at a diagnostic pathology laboratory. Cell proliferation assays were performed using the CyQuant assay as indicated above.

### Microarray analysis

HSC-3, tonsillar SCC, myoepithelial, and oral osteosarcoma cells were grown in 6-well plates on matrigel for 24 h. Cells were then treated with 1 µM LSD-GSK1 or vehicle (*n* = 4 biological replicates per condition) in respective groups for 48 h in serum-free media. Total RNA was isolated and subjected to microarray analysis. Affymetrix GeneChip Human Gene 2.0 ST CEL files were normalized to produce gene-level expression values using the implementation of the Robust Multiarray Average (RMA) [43] in the Affymetrix package (version 1.36.1) [44] included in the Bioconductor software suite (version 2.12) [45] and an Entrez Gene-specific probeset mapping (version 17.0.0) from the Molecular and Behavioral Neuroscience Institute (Brainarray) at the University of Michigan [46]. Array quality was assessed by computing Relative Log Expression (RLE) and Normalized Unscaled Standard Error (NUSE) using the affyPLM Bioconductor package (version 1.34.0). Pairwise differential expression was assessed using the moderated (empirical Bayesian) t-test implemented in the limma package (version 3.14.4) (i.e., creating simple linear models with lmFit, followed by empirical Bayesian adjustment with eBayes). A fold change of 2 indicates 2-fold higher expression in GSK-LSD1 treated cells relative to expression in vehicle treated cells. Correction for multiple hypothesis testing was accomplished using the Benjamini-Hochberg false discovery rate (FDR) and represented as FDR q values. All statistical analyses were performed using the R environment for statistical computing (version 2.15.1). Gene set enrichment analysis (GSEA) of the treatment signatures was performed using the pre-ranked GSEA functionality within the GSEA Desktop software (v2.2.2), with the t-test statistic of treatment vs no-treatment control for each cell line as the ranking variable. Gene sets queried obtained from MSigDB (http://software.broadinstitute.org/gsea/msigdb/).

### PDX model

Freshly isolated human tonsillar epithelial tumors were cut into small pieces using a 4 mm biopsy punch, mixed with Matrigel, and implanted on the back of nude mice to yield the P0 generation. Tumors were allowed to grow for 3 months after which they were transferred into nude mice (P1 generation). These P1 generation tumors were allowed to grow for 16 weeks in *n* = 20 mice. The mice were then divided into 2 groups: half of the mice were treated with vehicle (*n* = 10) and half with GSK-LSD1 (*n* = 10, 10 mg/kg) twice a week by subcutaneous injection near the tumor margin without damaging the tumor implant. The mice were sacrificed after 32 weeks of vehicle or GSK-LSD1 treatment, and tumor tissues were collected for RNA analyses.

### Immunofluorescence of human tissue sections with anti-CTGF

Human tissues from PDX models were fixed in 4% paraformaldehyde overnight, paraffin embedded, and sectioned. For immunofluorescence, the tissue sections were incubated with CTGF-specific antibodies (Abcam) or an isotype control antibody followed by biotin-conjugated secondary antibodies. The fluorescent signal was developed with streptavidin conjugated Texas Red. An anti-fade reagent was added to each sample prior to imaging. Quantification of images was performed using Image J software (NIH), as shown previously [47]. The fold-changes in expression compared to respective controls are indicated in the graph.

### Molecular analysis

Total RNA was extracted in Trizol according to the manufacturer’s instructions (Qiagen). Quantitative real-time PCR (RT-qPCR) analysis was performed using TaqMan gene expression assays from Life Technologies, according to a standard protocol [48].

## SUPPLEMENTARY MATERIALS FIGURES AND TABLES






