# Supplementary material for: Inhibition of LSD1 epigenetically attenuates oral cancer growth and metastasis
Source: Oncotarget. 2017 Jul 27;8(43):73372–86. doi: 10.18632/oncotarget.19637 (PMC5650269; doi:10.18632/oncotarget.19637)
Supplement: Supplementary file 1 [file oncotarget-08-73372-s001.pdf]

Inhibition of LSD1 epigenetically attenuates oral cancer growth and metastasis

SUPPLEMENTARY MATERIALS

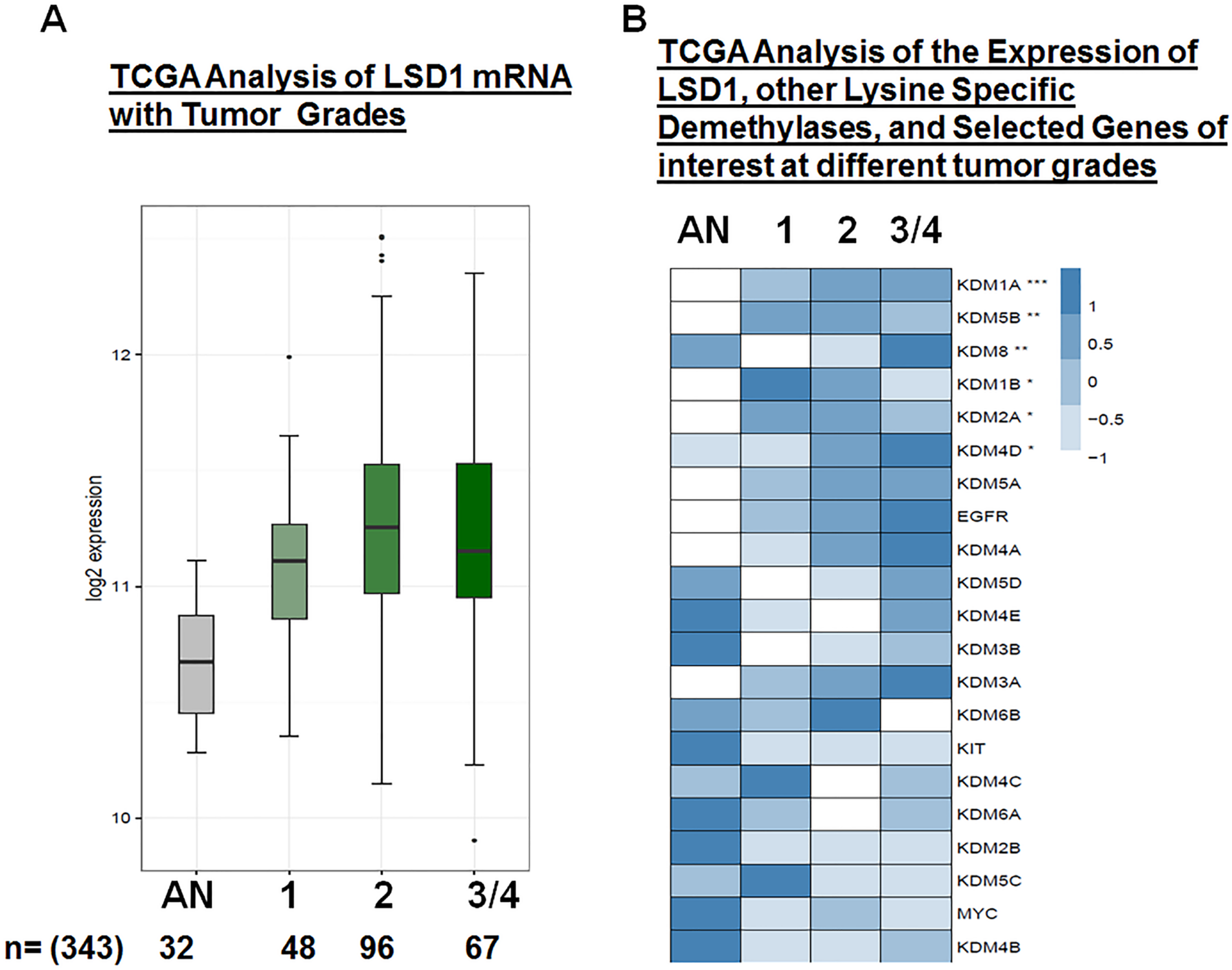

Supplementary Figure 1: LSD1 expression with tumor grades: A) TCGA analysis of the average LSD1 mRNA expression in different grade tumors compared to adjacent normal (AN) tongue tissue. **B**) A heatmap of the TCGA analysis showing average expression levels (row Z-scores) for the 21 genes of interest (ranked in increasing order of their FDR value, ***FDR<0.0001, **FDR<0.01 and *FDR<0.05) with respect to expression of LSD1.

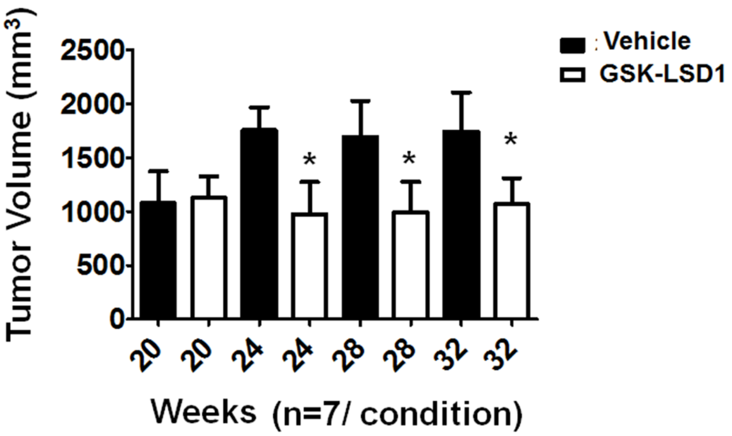

Supplementary Figure 2: GSK-LSD1 injection at 20 weeks inhibited the growth of pre-existing tumors in a PDX model (n=7 per condition). Statistical analyses were performed with unpaired Student's t-tests. * P-value<0.05.

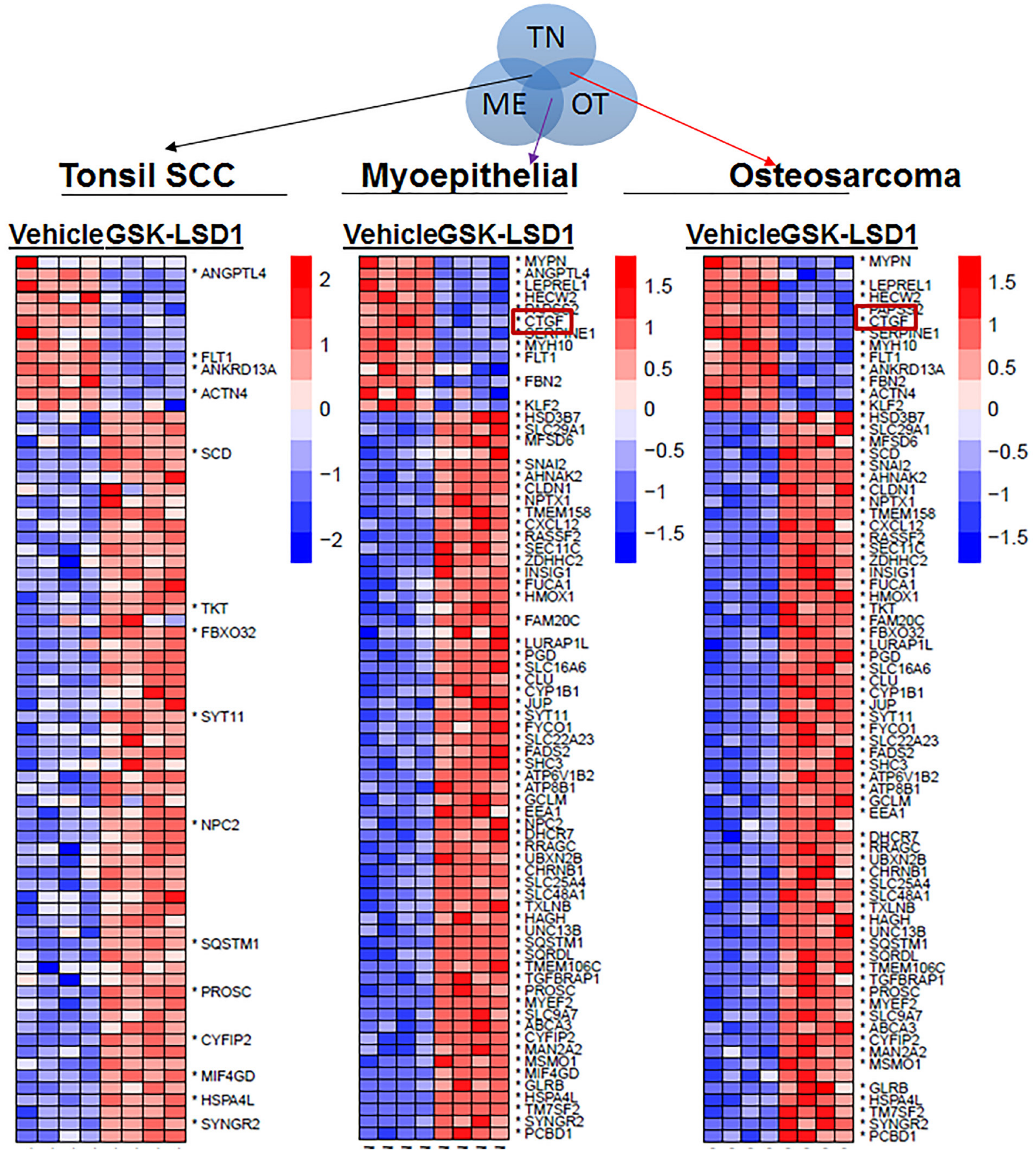

Supplementary Figure 3: Detailed gene signatures showing similarity in genes inhibited or activated by GSK-LSD1 compared to vehicle in patient-derived primary cells isolated from tumors.

For Supplementary Tables 1 and 2 See Supplementary Files
