# Supplementary material for: Inhibition of LSD1 epigenetically attenuates oral cancer growth and metastasis
Source: Oncotarget. 2017 Jul 27;8(43):73372–86. doi: 10.18632/oncotarget.19637 (PMC5650269; doi:10.18632/oncotarget.19637)
Supplement: Supplementary file 2 [file oncotarget-08-73372-s002.pdf]

FDR q-value	Name of Oncogenic signature
**Upreguated in TN**	
0.00E+00	CSR_LATE_UP.V1_DN
1.28E-03	RB_P107_DN.V1_DN
2.38E-03	SIRNA_EIF4GI_UP
3.53E-03	KRAS.DF.V1_UP
6.47E-03	AKT_UP.V1_UP
6.08E-03	RB_P130_DN.V1_DN
5.41E-03	RPS14_DN.V1_UP
5.07E-03	MTOR_UP.N4.V1_UP
5.14E-03	STK33_UP
4.94E-03	STK33_NOMO_UP
5.78E-03	PRC2_EZH2_UP.V1_DN
6.45E-03	BCAT.100_UP.V1_UP
8.70E-03	MTOR_UP.V1_UP
1.35E-02	MEL18_DN.V1_UP
1.27E-02	RB_DN.V1_DN
1.57E-02	P53_DN.V1_UP
1.76E-02	NFE2L2.V2
1.76E-02	AKT_UP_MTOR_DN.V1_UP
2.18E-02	TBK1.DN.48HRS_DN
2.19E-02	EIF4E_UP
2.37E-02	STK33_SKM_UP
2.39E-02	PIGF_UP.V1_UP
2.43E-02	ERB2_UP.V1_DN
2.71E-02	LEF1_UP.V1_DN
2.88E-02	LTE2_UP.V1_DN
3.08E-02	MEK_UP.V1_DN
3.11E-02	HOXA9_DN.V1_UP
3.39E-02	PTEN_DN.V2_UP
3.35E-02	STK33_NOMO_DN
4.39E-02	BMI1_DN.V1_DN
4.36E-02	ATF2_UP.V1_DN
4.28E-02	CAMP_UP.V1_UP
4.57E-02	BMI1_DN.V1_UP
4.81E-02	STK33_SKM_DN
4.99E-02	P53_DN.V1_DN
5.76E-02	EGFR_UP.V1_DN
8.95E-02	STK33_DN
9.43E-02	TBK1.DF_UP
9.47E-02	JNK_DN.V1_UP
1.11E-01	E2F1_UP.V1_DN
1.09E-01	HOXA9_DN.V1_DN
1.16E-01	MEK_UP.V1_UP
1.28E-01	BMI1_DN_MEL18_DN.V1_DN
1.27E-01	WNT_UP.V1_DN
1.45E-01	PTEN_DN.V1_DN
1.47E-01	KRAS.50_UP.V1_DN

1.49E-01	IL21_UP.V1_UP
1.52E-01	ESC_V6.5_UP_LATE.V1_UP
1.57E-01	ERB2_UP.V1_UP
1.70E-01	ALK_DN.V1_UP
1.73E-01	NOTCH_DN.V1_UP
1.85E-01	ESC_J1_UP_LATE.V1_UP
1.99E-01	KRAS.KIDNEY_UP.V1_DN
2.03E-01	GLI1_UP.V1_DN
2.02E-01	BMI1_DN_MEL18_DN.V1_UP
2.06E-01	KRAS.LUNG.BREAST_UP.V1_DN
2.02E-01	RB_P130_DN.V1_UP
2.01E-01	WNT_UP.V1_UP
2.00E-01	SNF5_DN.V1_UP
2.20E-01	IL15_UP.V1_DN
2.21E-01	AKT_UP.V1_DN
2.26E-01	KRAS.600.LUNG.BREAST_UP.V1_DN
2.32E-01	LTE2_UP.V1_UP
2.44E-01	BCAT_BILD_ET_AL_UP
2.43E-01	KRAS.LUNG_UP.V1_UP
2.39E-01	ATF2_S_UP.V1_DN
2.43E-01	CTIP_DN.V1_UP
2.49E-01	KRAS.300_UP.V1_DN
2.51E-01	TBK1.DN.48HRS_UP
**Downreguated in TN**	
6.34E-03	CORDENONSI_YAP_CONSERVED_SIGNATURE
4.68E-03	E2F1_UP.V1_UP
2.96E-02	PRC2_EDD_UP.V1_UP
4.71E-02	TGFB_UP.V1_UP
9.56E-02	EGFR_UP.V1_UP
1.28E-01	KRAS.PROSTATE_UP.V1_UP
1.43E-01	MYC_UP.V1_DN
1.45E-01	RAPA_EARLY_UP.V1_UP
2.13E-01	RPS14_DN.V1_DN
2.42E-01	GCNP_SHH_UP_LATE.V1_UP
2.39E-01	RAF_UP.V1_DN

Upregulated in ME

FDR q-val	NAME
0.00E+00	CSR_LATE_UP.V1_DN
0.00E+00	ATF2_UP.V1_DN
0.00E+00	STK33_UP
2.92E-04	PIGF_UP.V1_UP
1.16E-03	SIRNA_EIF4GI_UP
1.17E-03	MTOR_UP.N4.V1_UP
9.99E-04	STK33_NOMO_UP
1.16E-03	RPS14_DN.V1_UP
1.17E-03	RB_P107_DN.V1_DN
1.28E-03	MEK_UP.V1_DN
1.37E-03	RB_P130_DN.V1_DN

1.35E-03	HOXA9_DN.V1_UP
1.25E-03	ATF2_S_UP.V1_DN
1.16E-03	LTE2_UP.V1_UP
1.38E-03	PRC2_EZH2_UP.V1_DN
1.44E-03	STK33_SKM_UP
1.62E-03	MEL18_DN.V1_UP
1.65E-03	P53_DN.V1_UP
1.87E-03	RAF_UP.V1_UP
2.00E-03	BMI1_DN.V1_UP
2.07E-03	EGFR_UP.V1_UP
2.83E-03	P53_DN.V1_DN
2.89E-03	EIF4E_DN
2.77E-03	STK33_DN
2.80E-03	BMI1_DN_MEL18_DN.V1_UP
2.69E-03	TBK1.DF_DN
2.59E-03	ESC_J1_UP_LATE.V1_UP
2.79E-03	CSR_EARLY_UP.V1_DN
3.75E-03	TBK1.DN.48HRS_UP
4.37E-03	STK33_NOMO_DN
4.34E-03	ESC_J1_UP_EARLY.V1_UP
5.05E-03	PRC2_EDD_UP.V1_DN
4.93E-03	LEF1_UP.V1_DN
4.82E-03	EGFR_UP.V1_DN
4.75E-03	STK33_SKM_DN
4.98E-03	TBK1.DF_UP
7.25E-03	CAMP_UP.V1_DN
7.87E-03	KRAS.KIDNEY_UP.V1_UP
8.20E-03	TBK1.DN.48HRS_DN
9.00E-03	CAMP_UP.V1_UP
8.97E-03	KRAS.DF.V1_UP
1.05E-02	SNF5_DN.V1_DN
1.18E-02	JNK_DN.V1_UP
1.60E-02	NFE2L2.V2
2.29E-02	ERB2_UP.V1_DN
2.29E-02	EIF4E_UP
2.25E-02	KRAS.600_UP.V1_UP
2.23E-02	AKT_UP_MTOR_DN.V1_UP
2.19E-02	RB_DN.V1_DN
2.16E-02	HOXA9_DN.V1_DN
2.47E-02	MEK_UP.V1_UP
2.47E-02	ERB2_UP.V1_UP
2.45E-02	E2F1_UP.V1_DN
2.41E-02	BCAT_BILD_ET_AL_UP
2.65E-02	YAP1_UP
2.61E-02	BMI1_DN.V1_DN
3.36E-02	PDGF_ERK_DN.V1_UP
3.34E-02	PTEN_DN.V2_UP
4.10E-02	BCAT_GDS748_DN
4.09E-02	KRAS.300_UP.V1_UP

4.61E-02	MTOR_UP.N4.V1_DN
4.97E-02	VEGF_A_UP.V1_UP
5.58E-02	MTOR_UP.V1_UP
5.59E-02	IL15_UP.V1_DN
6.73E-02	KRAS.50_UP.V1_UP
7.73E-02	IL2_UP.V1_DN
7.75E-02	RAF_UP.V1_DN
9.55E-02	AKT_UP.V1_DN
9.88E-02	MTOR_UP.V1_DN
1.17E-01	BCAT.100_UP.V1_UP
1.49E-01	SIRNA_EIF4GI_DN
1.58E-01	P53_DN.V2_DN
1.57E-01	CAHOY_ASTROGLIAL
1.81E-01	BMI1_DN_MEL18_DN.V1_DN
1.81E-01	GLI1_UP.V1_DN
1.85E-01	VEGF_A_UP.V1_DN
1.85E-01	LEF1_UP.V1_UP
1.84E-01	AKT_UP.V1_UP
1.84E-01	IL15_UP.V1_UP
1.83E-01	NOTCH_DN.V1_UP
1.91E-01	ESC_V6.5_UP_LATE.V1_UP
1.97E-01	ALK_DN.V1_UP
2.08E-01	WNT_UP.V1_DN
2.12E-01	ATF2_S_UP.V1_UP
2.19E-01	JAK2_DN.V1_DN
2.29E-01	PRC2_SUZ12_UP.V1_DN
2.40E-01	ESC_J1_UP_EARLY.V1_DN
**Downregulated in ME**	
1.85E-01	TGFB_UP.V1_UP
1.73E-01	CORDENONSI_YAP_CONSERVED_SIGNATURE
1.28E-01	CSR_LATE_UP.V1_UP
1.02E-01	ALK_DN.V1_DN
1.39E-01	CSR_EARLY_UP.V1_UP
1.41E-01	DCA_UP.V1_UP
1.38E-01	ESC_J1_UP_LATE.V1_DN
1.94E-01	ESC_V6.5_UP_EARLY.V1_DN
1.91E-01	RB_P107_DN.V1_UP
1.92E-01	PRC2_SUZ12_UP.V1_UP
1.91E-01	NRL_DN.V1_UP
2.07E-01	IL2_UP.V1_UP

Upregulated in OT

FDR q-val	NAME
0.00E+00	ATF2_UP.V1_DN
0.00E+00	CSR_LATE_UP.V1_DN
4.24E-04	PRC2_EZH2_UP.V1_DN
5.45E-03	ERB2_UP.V1_UP
5.22E-03	BCAT.100_UP.V1_UP
4.35E-03	NFE2L2.V2

7.39E-03	EIF4E_DN
8.77E-03	P53_DN.V1_DN
8.54E-03	PRC2_EDD_UP.V1_DN
1.18E-02	RPS14_DN.V1_UP
1.36E-02	RB_P130_DN.V1_DN
1.30E-02	MEK_UP.V1_DN
1.22E-02	ATF2_S_UP.V1_DN
1.32E-02	SIRNA_EIF4GI_UP
1.40E-02	HOXA9_DN.V1_UP
1.49E-02	VEGF_A_UP.V1_UP
1.47E-02	MEK_UP.V1_UP
1.38E-02	SINGH_KRAS_DEPENDENCY_SIGNATURE_
1.37E-02	MTOR_UP.N4.V1_UP
1.56E-02	PRC2_SUZ12_UP.V1_DN
1.50E-02	KRAS.50_UP.V1_UP
1.61E-02	AKT_UP.V1_UP
1.86E-02	KRAS.600.LUNG.BREAST_UP.V1_DN
2.01E-02	BMI1_DN.V1_DN
2.12E-02	MEL18_DN.V1_DN
2.36E-02	LTE2_UP.V1_UP
2.39E-02	ESC_J1_UP_EARLY.V1_UP
2.67E-02	E2F1_UP.V1_DN
2.80E-02	RAF_UP.V1_UP
3.25E-02	KRAS.LUNG.BREAST_UP.V1_DN
3.14E-02	IL21_UP.V1_DN
4.02E-02	MTOR_UP.V1_UP
4.61E-02	YAP1_DN
4.49E-02	STK33_SKM_DN
5.43E-02	IL15_UP.V1_DN
5.47E-02	BCAT_GDS748_DN
5.43E-02	LEF1_UP.V1_DN
5.32E-02	KRAS.KIDNEY_UP.V1_UP
6.52E-02	STK33_NOMO_UP
6.37E-02	KRAS.BREAST_UP.V1_DN
6.63E-02	STK33_UP
6.73E-02	JNK_DN.V1_UP
7.38E-02	KRAS.LUNG_UP.V1_DN
7.39E-02	STK33_NOMO_DN
7.49E-02	PTEN_DN.V2_UP
7.32E-02	MEL18_DN.V1_UP
7.63E-02	STK33_SKM_UP
8.07E-02	AKT_UP_MTOR_DN.V1_DN
8.33E-02	IL2_UP.V1_DN
8.42E-02	AKT_UP_MTOR_DN.V1_UP
8.42E-02	STK33_DN
8.30E-02	BCAT.100_UP.V1_DN
8.44E-02	BMI1_DN_MEL18_DN.V1_DN
8.82E-02	CTIP_DN.V1_UP
9.07E-02	P53_DN.V1_UP

8.92E-02	RB_DN.V1_DN
1.02E-01	JAK2_DN.V1_UP
1.02E-01	KRAS.300_UP.V1_DN
1.17E-01	NOTCH_DN.V1_UP
1.19E-01	AKT_UP.V1_DN
1.34E-01	KRAS.600_UP.V1_DN
1.38E-01	PIGF_UP.V1_UP
1.48E-01	MTOR_UP.V1_DN
1.54E-01	IL21_UP.V1_UP
1.73E-01	CYCLIN_D1_KE_.V1_DN
1.75E-01	CSR_EARLY_UP.V1_DN
1.75E-01	KRAS.DF.V1_UP
1.93E-01	PKCA_DN.V1_UP
1.95E-01	TGFB_UP.V1_DN
2.05E-01	KRAS.50_UP.V1_DN
2.12E-01	EGFR_UP.V1_DN
2.14E-01	KRAS.600_UP.V1_UP
2.22E-01	PRC1_BMI_UP.V1_DN
2.22E-01	LTE2_UP.V1_DN
2.26E-01	RB_P107_DN.V1_DN
2.33E-01	CAHOY_ASTROCYTIC
2.55E-01	NOTCH_DN.V1_DN

Downregulated in OT

FDR q-val	Name
0.00E+00	CORDENONSI_YAP_CONSERVED_SIGNATURE
0.00E+00	CSR_LATE_UP.V1_UP
2.20E-04	E2F1_UP.V1_UP
1.65E-04	CSR_EARLY_UP.V1_UP
2.86E-04	RB_P107_DN.V1_UP
2.38E-04	RPS14_DN.V1_DN
1.17E-03	SIRNA_EIF4GI_DN
2.32E-03	VEGF_A_UP.V1_DN
2.31E-03	ESC_V6.5_UP_EARLY.V1_DN
3.50E-03	HINATA_NFKB_IMMU_INF
3.59E-03	GCNP_SHH_UP_EARLY.V1_UP
5.71E-03	BMI1_DN_MEL18_DN.V1_UP
6.11E-03	TGFB_UP.V1_UP
8.34E-03	PRC2_EZH2_UP.V1_UP
8.88E-03	PDGF_ERK_DN.V1_DN
1.05E-02	ESC_J1_UP_EARLY.V1_DN
1.96E-02	GCNP_SHH_UP_LATE.V1_UP
3.62E-02	PDGF_UP.V1_UP
3.57E-02	ESC_J1_UP_LATE.V1_UP
