# Supplementary material for: Inhibition of LSD1 epigenetically attenuates oral cancer growth and metastasis
Source: Oncotarget. 2017 Jul 27;8(43):73372–86. doi: 10.18632/oncotarget.19637 (PMC5650269; doi:10.18632/oncotarget.19637)
Supplement: Supplementary file 3 [file oncotarget-08-73372-s003.pdf]

| FDR q-value | Name of hallmark geneset |
|---|---|
| **Upregulated in TN** | |
| 0.00E+00 | HALLMARK_CHOLESTEROL_HOMEOSTASIS |
| 0.00E+00 | HALLMARK_MTORC1_SIGNALING |
| 0.00E+00 | HALLMARK_OXIDATIVE_PHOSPHORYLATION |
| 0.00E+00 | HALLMARK_INTERFERON_ALPHA_RESPONSE |
| 6.78E-04 | HALLMARK_BILE_ACID_METABOLISM |
| 5.65E-04 | HALLMARK_REACTIVE_OXIGEN_SPECIES_PATHWAY |
| 4.85E-04 | HALLMARK_P53_PATHWAY |
| 4.24E-04 | HALLMARK_INTERFERON_GAMMA_RESPONSE |
| 3.77E-04 | HALLMARK_UV_RESPONSE_UP |
| 3.39E-04 | HALLMARK_XENOBIOTIC_METABOLISM |
| 2.04E-03 | HALLMARK_PEROXISOME |
| 2.73E-03 | HALLMARK_APOPTOSIS |
| 3.46E-03 | HALLMARK_ESTROGEN_RESPONSE_LATE |
| 3.50E-03 | HALLMARK_FATTY_ACID_METABOLISM |
| 3.27E-03 | HALLMARK_ADIPOGENESIS |
| 4.53E-03 | HALLMARK_ANDROGEN_RESPONSE |
| 7.30E-03 | HALLMARK_PROTEIN_SECRETION |
| 8.19E-03 | HALLMARK_MYOGENESIS |
| 1.71E-02 | HALLMARK_IL6_JAK_STAT3_SIGNALING |
| 1.65E-02 | HALLMARK_COAGULATION |
| 1.68E-02 | HALLMARK_HEME_METABOLISM |
| 1.77E-02 | HALLMARK_MYC_TARGETS_V1 |
| 1.69E-02 | HALLMARK_COMPLEMENT |
| 3.51E-02 | HALLMARK_ESTROGEN_RESPONSE_EARLY |
| 5.04E-02 | HALLMARK_EPITHELIAL_MESENCHYMAL_TRANSITION |
| 6.08E-02 | HALLMARK_TNFA_SIGNALING_VIA_NFKB |
| 6.72E-02 | HALLMARK_KRAS_SIGNALING_UP |
| 6.62E-02 | HALLMARK_GLYCOLYSIS |
| 1.16E-01 | HALLMARK_IL2_STAT5_SIGNALING |
| 1.23E-01 | HALLMARK_UNFOLDED_PROTEIN_RESPONSE |
| 1.30E-01 | HALLMARK_INFLAMMATORY_RESPONSE |
| 1.74E-01 | HALLMARK_ANGIOGENESIS |
| **Downregulated in TN** | |
| 3.48E-02 | HALLMARK_MITOTIC_SPINDLE |
| 2.25E-02 | HALLMARK_UV_RESPONSE_DN |
| 1.60E-02 | HALLMARK_APICAL_JUNCTION |
| 2.81E-02 | HALLMARK_MYC_TARGETS_V2 |
| 5.77E-02 | HALLMARK_E2F_TARGETS |
| 9.82E-02 | HALLMARK_G2M_CHECKPOINT |
| 1.14E-01 | HALLMARK_HYPOXIA |
| | |
| **Upregualted in ME** | |
| 0.00E+00 | HALLMARK_OXIDATIVE_PHOSPHORYLATION |
| 0.00E+00 | HALLMARK_CHOLESTEROL_HOMEOSTASIS |
| 0.00E+00 | HALLMARK_MTORC1_SIGNALING |

| | |
|---|---|
| 0.00E+00 | HALLMARK_REACTIVE_OXIGEN_SPECIES_PATHWAY |
| 0.00E+00 | HALLMARK_PROTEIN_SECRETION |
| 0.00E+00 | HALLMARK_ADIPOGENESIS |
| 0.00E+00 | HALLMARK_HEME_METABOLISM |
| 4.51E-04 | HALLMARK_BILE_ACID_METABOLISM |
| 4.01E-04 | HALLMARK_MYOGENESIS |
| 1.66E-03 | HALLMARK_FATTY_ACID_METABOLISM |
| 1.82E-03 | HALLMARK_P53_PATHWAY |
| 2.84E-03 | HALLMARK_PEROXISOME |
| 3.08E-03 | HALLMARK_KRAS_SIGNALING_UP |
| 3.44E-03 | HALLMARK_ESTROGEN_RESPONSE_LATE |
| 4.96E-03 | HALLMARK_APOPTOSIS |
| 4.86E-03 | HALLMARK_EPITHELIAL_MESENCHYMAL_TRANSITION |
| 4.85E-03 | HALLMARK_COAGULATION |
| 5.22E-03 | HALLMARK_XENOBIOTIC_METABOLISM |
| 1.18E-02 | HALLMARK_COMPLEMENT |
| 1.83E-02 | HALLMARK_UV_RESPONSE_DN |
| 1.80E-02 | HALLMARK_ESTROGEN_RESPONSE_EARLY |
| 1.94E-02 | HALLMARK_DNA_REPAIR |
| 2.02E-02 | HALLMARK_UNFOLDED_PROTEIN_RESPONSE |
| 3.21E-02 | HALLMARK_PI3K_AKT_MTOR_SIGNALING |
| 3.12E-02 | HALLMARK_ANGIOGENESIS |
| 5.10E-02 | HALLMARK_UV_RESPONSE_UP |
| 4.93E-02 | HALLMARK_APICAL_SURFACE |
| 4.98E-02 | HALLMARK_MYC_TARGETS_V1 |
| 6.92E-02 | HALLMARK_ANDROGEN_RESPONSE |
| 8.27E-02 | HALLMARK_IL2_STAT5_SIGNALING |
| 9.34E-02 | HALLMARK_GLYCOLYSIS |
| 1.98E-01 | HALLMARK_INFLAMMATORY_RESPONSE |
| **Downregulated in ME** | |
| 1.49E-03 | HALLMARK_E2F_TARGETS |
| 2.03E-03 | HALLMARK_G2M_CHECKPOINT |
| 1.36E-03 | HALLMARK_WNT_BETA_CATENIN_SIGNALING |
| 1.60E-01 | HALLMARK_TNFA_SIGNALING_VIA_NFKB |
| 1.64E-01 | HALLMARK_MITOTIC_SPINDLE |
| 2.34E-01 | HALLMARK_HYPOXIA |
| | |
| **Upregualted in OT** | |
| 0.00E+00 | HALLMARK_INTERFERON_ALPHA_RESPONSE |
| 0.00E+00 | HALLMARK_INTERFERON_GAMMA_RESPONSE |
| 0.00E+00 | HALLMARK_CHOLESTEROL_HOMEOSTASIS |
| 7.36E-04 | HALLMARK_XENOBIOTIC_METABOLISM |
| 9.04E-04 | HALLMARK_P53_PATHWAY |
| 9.26E-04 | HALLMARK_BILE_ACID_METABOLISM |
| 7.94E-04 | HALLMARK_REACTIVE_OXIGEN_SPECIES_PATHWAY |
| 4.55E-03 | HALLMARK_COAGULATION |
| 5.92E-03 | HALLMARK_FATTY_ACID_METABOLISM |

| | |
|---|---|
| 5.48E-03 | HALLMARK_ESTROGEN_RESPONSE_LATE |
| 6.57E-03 | HALLMARK_MYOGENESIS |
| 6.67E-03 | HALLMARK_APOPTOSIS |
| 6.89E-03 | HALLMARK_UV_RESPONSE_UP |
| 6.48E-03 | HALLMARK_HEME_METABOLISM |
| 8.08E-03 | HALLMARK_MTORC1_SIGNALING |
| 1.11E-02 | HALLMARK_COMPLEMENT |
| 1.23E-02 | HALLMARK_ADIPOGENESIS |
| 1.25E-02 | HALLMARK_KRAS_SIGNALING_UP |
| 2.34E-02 | HALLMARK_ESTROGEN_RESPONSE_EARLY |
| 3.09E-02 | HALLMARK_IL2_STAT5_SIGNALING |
| 4.57E-02 | HALLMARK_PEROXISOME |
| 9.33E-02 | HALLMARK_ANGIOGENESIS |
| 1.11E-01 | HALLMARK_IL6_JAK_STAT3_SIGNALING |
| 1.43E-01 | HALLMARK_APICAL_SURFACE |
| 1.47E-01 | HALLMARK_PROTEIN_SECRETION |
| 1.72E-01 | HALLMARK_OXIDATIVE_PHOSPHORYLATION |
| 1.95E-01 | HALLMARK_ANDROGEN_RESPONSE |
| 1.98E-01 | HALLMARK_KRAS_SIGNALING_DN |
| **Downregulated in OT** | |
| 0.00E+00 | HALLMARK_E2F_TARGETS |
| 0.00E+00 | HALLMARK_G2M_CHECKPOINT |
| 0.00E+00 | HALLMARK_MYC_TARGETS_V1 |
| 0.00E+00 | HALLMARK_MYC_TARGETS_V2 |
| 1.38E-04 | HALLMARK_MITOTIC_SPINDLE |
| 3.37E-03 | HALLMARK_HYPOXIA |
| 4.98E-03 | HALLMARK_UV_RESPONSE_DN |
| 6.41E-03 | HALLMARK_UNFOLDED_PROTEIN_RESPONSE |
| 8.88E-03 | HALLMARK_EPITHELIAL_MESENCHYMAL_TRANSITION |
| 8.51E-03 | HALLMARK_TNFA_SIGNALING_VIA_NFKB |
| 2.40E-02 | HALLMARK_APICAL_JUNCTION |
| 5.91E-02 | HALLMARK_DNA_REPAIR |
